# Automatic Cephalometric Landmark Identification System Based on the Multi-Stage Convolutional Neural Networks with CBCT Combination Images

**DOI:** 10.3390/s21020505

**Published:** 2021-01-12

**Authors:** Min-Jung Kim, Yi Liu, Song Hee Oh, Hyo-Won Ahn, Seong-Hun Kim, Gerald Nelson

**Affiliations:** 1 Department of Orthodontics, Graduate School, Kyung Hee University, Seoul 02447, Korea; grace206@naver.com (M.-J.K.); hyowon@khu.ac.kr (H.-W.A.); 2 Department of Orthodontics, Peking University School of Stomatology, Beijing 100081, China; lyortho@163.com; 3 Department of Oral and Maxillofacial Radiology, Graduate School, Kyung Hee University, Seoul 02447, Korea; ohbbang50@gmail.com; 4 Division of Orthodontics, Department of Orofacial Science, University of California San Francisco, San Francisco, CA 94143, USA; gdnelson41@gmail.com

**Keywords:** artificial intelligence, convolutional neural networks, automatic identification, lateral cephalograms, cone-beam computed tomography, maximum intensity projection (MIP)

## Abstract

This study was designed to develop and verify a fully automated cephalometry landmark identification system, based on multi-stage convolutional neural networks (CNNs) architecture, using a combination dataset. In this research, we trained and tested multi-stage CNNs with 430 lateral and 430 MIP lateral cephalograms synthesized by cone-beam computed tomography (CBCT) to make a combination dataset. Fifteen landmarks were manually and respectively identified by experienced examiner, at the preprocessing phase. The intra-examiner reliability was high (ICC = 0.99) in manual identification. The results of prediction of the system for average mean radial error (MRE) and standard deviation (SD) were 1.03 mm and 1.29 mm, respectively. In conclusion, different types of image data might be the one of factors that affect the prediction accuracy of a fully-automated landmark identification system, based on multi-stage CNNs.

## 1. Introduction 

As a prerequisite for diagnosis in orthodontic treatment, cephalometric analysis is examined with a goal to achieve a much higher accuracy [1]. The conventional cephalogram is most used in orthodontics, however, it only provides plane information from a three-dimensional (3D) craniofacial structure [2]. Emergence of the cone-beam computed tomography (CBCT) provides high-quality diagnostic images to develop an appropriate treatment plan and facilitate successful orthodontic and orthognathic treatment results [3,4]. Typical software programs enable the clinician to synthesize two-dimensional (2D) digital radiographs in multi-angles with various algorithms, from the CBCT image [3,4]. Several images can be synthesized from one CBCT scan and used for 2D or 3D cephalometric analysis [5,6,7].

A 2D radiograph synthesized from the CBCT has the advantages of maintaining the object size without magnification, and the ability to adjust the head position to reduce distortion (reorientation) [8,9,10,11]. Maximum intensity projection (MIP) is one of the image modalities available in software used to synthesize 2D from CBCT. The visualization effect made with maximum intensity issues voxels parallel on the projection plane. The CBCT image is displayed in a matrix of isotropic voxels or volume elements. The concept of the pixel is 2D cross-sections of voxels that represent image density, or the absorption features of a specific structure in the CBCT image. The highest value of the pixel column corresponds to the projection displaying the anatomic structure with clearer contours. Therefore, the 3D craniofacial image orthographically projected onto the plane to synthesize the 2D MIP lateral cephalogram, has the advantage of a clearer facial tissue profile, an important analytic feature of orthodontic cephalometry [6,12].

Several studies propose systems of fully automated cephalometry landmark identification, based on a machine learning technique [13,14,15,16,17]. Deep learning is subset of the machine learning concept that gives outstanding abilities to recognize features of a complicated image [18,19,20,21]. Several factors influence the accuracy of landmark identification with deep learning—the type of deep learning architecture, the number of datasets, the image quality, or the number of landmarks and identification pattern. Some studies exist used conventional lateral cephalograms and a deep learning architecture based on a fully automated cephalometry landmark identification system. They report a precision range of 2 mm [22,23,24,25].

Accordingly, lateral cephalograms synthesized by CBCT are able to minimize intervention in the layered structure images and enable us to easily identify landmarks. It also has less distortion images than 2D conventional lateral cephalograms. As mentioned above, MIP is able to intensively and clearly address the skeletal structure. Therefore, we assumed that the accuracy of automatic landmark identification would increase if both kinds of lateral cephalograms are used to take advantages of. 

This study was designed to make a combination dataset that consisted of 2D lateral and MIP lateral cephalograms synthesized by CBCT, to perform efficient landmark identification. It was made for developing and verifying a fully automated cephalometry landmark identification system, based on the multi-stage convolutional neural networks (CNNs) architecture.

## 2. Materials and Methods 

This retrospective study was performed under approval from the Institutional Review Board of Kyung Hee University Dental Hospital (IRB Number: IRB-KH DT19013). Informed consent was waived due to the retrospective nature of this study. All experiments were carried out in accordance with the approved guidelines.

### 2.1. Subjects 

Subjects were randomly selected from the PACS (picture archiving and communication systems) database at Kyung Hee University Dental Hospital. Subjects that interfered with landmarks identification with missing upper and lower permanent incisors, missing permanent upper and lower first molars, craniofacial syndromes, or dento-facial traumas, were excluded. All age, gender, and skeletal discrepancy were included. A total of 430 CBCT scans were selected for CBCT-LC and MIP-LC.

### 2.2. Image Acquisition from CBCT

#### 2.2.1. CBCT Protocol 

The CBCT scans were taken with a 0.39-mm^3^ voxel size level, 16 × 13 cm field of view, 10 mA, 80 kV, and 30 s scan time (Alphad vega, Asahi Roentgen Inc., Kyoto, Japan). The obtained data were imported as DICOM (Digital Imaging and Communications in Medicine) files to the Dolphin software 11.95 Premium (Dolphin Imaging & Management Solutions, Chatsworth, CA, USA). 

#### 2.2.2. Reorientation 

The 430 CBCT images were oriented according to the anatomic structures of reference [26]. The horizontal plane was established using the skeletal orbitale and right porion. The sagittal plane passing through the nasion was perpendicular to the horizontal plane. The coronal plane passing through nasion, perpendicular to the horizontal plane and sagittal plane, was utilized to finish the reorientation. Simultaneously, we aligned the bilateral fronto-zygomatic point to the same coordinate of X-axis in the lateral view. The consistent coordination was used for all CBCT images. 

#### 2.2.3. Synthesizing the Cephalogram 

**Figure 1:**
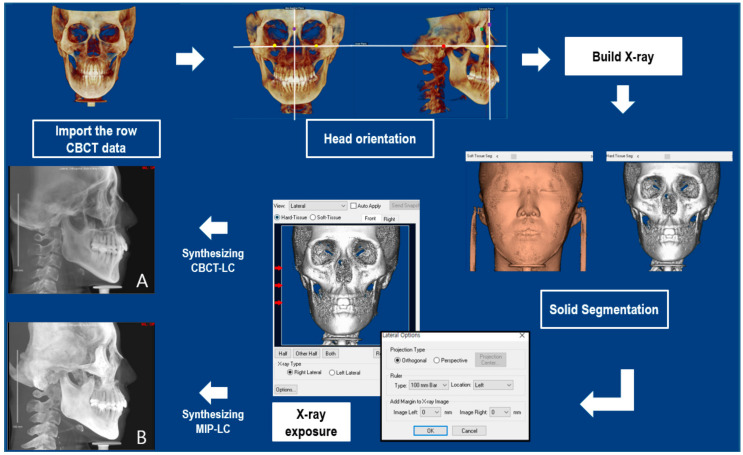
Image acquisition from CBCT: Import the CBCT data to the Dolphin software and reorient the head image. Using the ‘Build X-ray’ function in the software to synthesize the CBCT lateral cephalogram (CBCT-LC) and CBCT MIP lateral cephalogram (MIP-LC) with orthogonal X-ray. All synthesized image data were saved with a range of pixel size width at 2048 pix and the height at 1755–1890 pix in the JPG format.

A total of 430 reoriented CBCT images were used to automatically synthesize the CBCT-LC and MIP-LC by the Dolphin software, to prepare the dataset (Figure 1). A total of 860 images were prepared for multi-stage CNN training and testing. Based on previous findings [27,28], 80% of the data were prepared for deep CNNs training and 20% of data were used for testing, or 345 and 85 for each dataset. The synthesized images were saved with a range of pixel size width at 2048 pix, and height at 1755–1890 pix in JPG format.

### 2.3. Reproducibility of Intra-Examiner 

We randomly selected 50 CBCT-LC and 50 MIP-LC to verify the inter-examiner reproducibility. All landmarks were identified twice at intervals of two weeks, by a single examiner (MJK). The See-through Ceph (See-through Tech Inc., Seoul, Korea) software was implemented to accomplish landmark identification. Pixel values of each landmark were extracted in an Excel file (version 2010; Microsoft, Redmond, Washington). The intraclass-correlation-coefficient (ICC) was calculated to indicate reproducibility in intra-examiner repetitive identification with 95% confidence intervals. Statistical analyses were performed using the SPSS software (version-22.0, SPSS Inc., Chicago, IL, USA).

### 2.4. Multi-Stage CNNs Architecture

The multi-stage CNNs used in this study was developed with customized open-source Keras library and TensorFlow (Google, Mountain View, CA, USA), backend on Python language. The computer components used in this study were—CPU: AMD Ryzen 7 1700; Mainboard: MSI Z370-A PRO; Ram: SAMSUNG DDR4 16G x 2; GPU: Geforce, GTX 1080Ti; POWER: Master Watt Lite 600 80PLUS Standard 230V EU; SSD: SAMSUNG 850 EVO Series 256 GB; and Linux: Ubuntu 14.04 (Canonical, London, UK). 

One of the prominent abilities in deep CNNs learning is that it disseminates salient feature information based on a hierarchy to the subsequent layers. CNNs’ architecture is composed of convolution layers, pooling, and dense layers (fully connected layer). The target features of image were extracted from the convolution layers and pooling, during training. At the convolution layer, two-dimensional simple addition and multiplication were performed using suitable filters. Convolution operation outputs the intuitive measurement of the spatial similarity of the input. The CNNs learns from a filter that observes a specific image pattern in some spatial location of the previous layer’s output. The pooling layer is to sort out sampling and to prevent the number of parameters from increasing gradually. Commonly used pooling operation in CNNs is maximum pooling, which takes the maximum value pixels. Last layers of CNNs are dense layers, where a set of neurons are fully connected and the feature of input data to the network is classified to make a final decision [18,19]. 

At the image preprocessing stage, the first input model was developed with 400 widths, 400 height, and three-color channels—R (red), G (green), B (blue). In the model architecture, six convolutional layers extracted target features from the input image. 

Mathematically, a convolution of two functions ‘*f*’ and ‘*g*’ was defined as:

(1) $$\left(f*g\right)\left(i\right)=\overset{m}{\underset{j=1}{\sum }}g\left(j\right)\cdot f\left(i-j+\frac{m}{2}\right)$$

**Figure 2:**
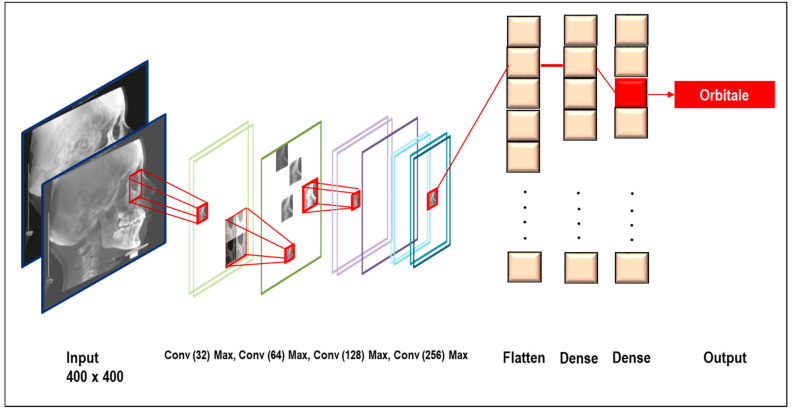
Schematic diagram of our proposed multi-stage CNNs architecture. Put the preprocessed image data. Input data passing through the deep CNNs model of composition with 6 convolution layers, with 32 × 2, 64 × 2, 128, 256 nodes, and max pooling for the features to be extracted. Subsequently, the two dense layers classified the features to make final decision.

**Figure 3:**
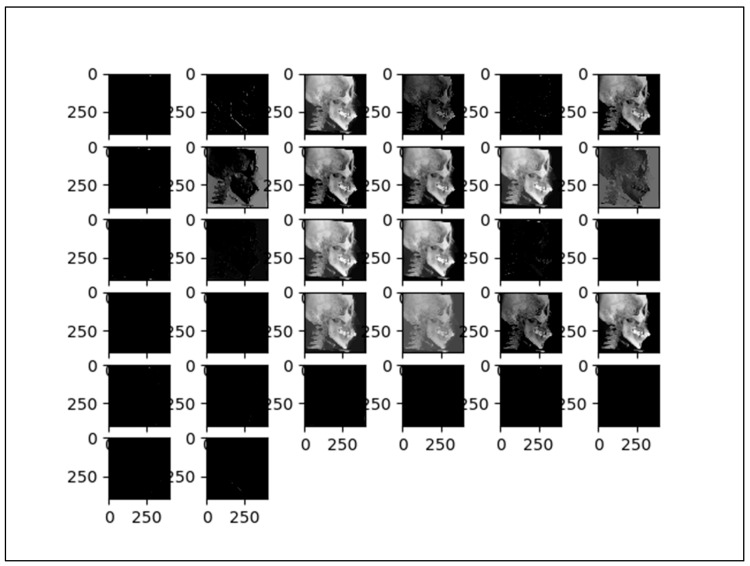
The visualized effect of input data at convolution layers during training.

Therefore, the algorithm could only be expressed by the dot products in the input function and a kernel function, which we used. A unit conversion used 1 mm = 10 pixels. To increase the learning effect, data augmentation techniques were applied as follows—up to left, right, up and down 50 pixels shift, up to 10° rotation left and right each side. To input the training dataset, the deep CNNs must first learn the full image. At a second learning phase, each 15 landmarks on the lateral cephalograms was cropped and trained with different sizes—250, 200, 150, 100, and 50, so that a total of five stages of multiple convolutional layers were arranged in parallel. Fifteen landmarks used for the Tweemac cephalometric analysis were to be intensively trained by CNNs [29]. The definition of 15 landmarks are described in Table 1. Schematic diagram of our proposed multi-stage CNNs architecture is in Figure 2, and the visualization is in Figure 3.

### 2.5. System Evaluation 

The accuracy of AI prediction was evaluated by mean radial errors (MRE) and a successful detection rate (SDR). MRE (mm) was the absolute distance differences between the manual identification (truth ground) and the AI prediction position. Definition:

(2) $$MRE\;=\;\frac{\sum_{i=1}^{n}R_{1}}{n}\;(mm)$$

Standard deviation (SD) = ${\sqrt{\frac{\sum_{i=1}^{n}\left(R_{i}-MRE\right)}{n-1}}}^{2}$, R = $\sqrt{{\Delta x}^{2}+{\Delta y}^{2}}$.

SDR (%) represents percentages of the absolute landmark distance difference between manual identification and the AI prediction position, if the prediction range was less than 2 mm, it was considered to be the clinical acceptance level.

(3) $$SDR\;(\%)\;=\;\frac{number\;of\;accurate\;identification}{number\;of\;identification}\times 100\%$$

Common ranges of ≤2 mm, 2.5 mm, 3 mm, 4 mm were used to divide the groups for the number of accurate identification in SDR. 

### 2.6. AI Prediction on Different Lateral Cephalograms 

The paired *t*-test was used to compare the AI prediction on CBCT-LC and MIP-LC. To verify whether AI could make better prediction on MIP-LC or not, statistical analysis was performed using the SPSS software (version 22.0; IBM, Armonk, NY, USA). Measurements were calculated and statistically analyzed at the 0.05 level of significance.

## 3. Results

The ICC was 0.99, which had high rate of reproducibility in intra-examiner repetitive identification. 

**Figure 4:**
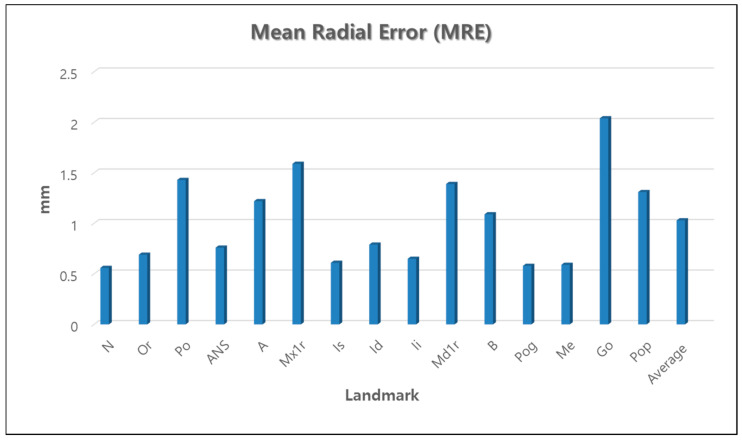
Mean Radial Error of each landmark and average MRE.

The results of AI prediction with the combination data showed an average MRE of 1.03 ± 1.29 mm, and SDR of 2.0 mm, 2.5 mm, 3.0 mm, and 4.0 mm precision ranges achieved 87.13%, 91.19%, 93.52%, 96.59%, respectively. The details between manual identification and AI prediction for each landmark are described in Table 2 and Figure 4. 

The MRE for each landmark revealed that nasion showed the highest accuracy, and gonion showed the lowest. Eight landmarks out of the nineteen yielded distance errors ranging within 1 mm. Six landmarks showed accurate MRE range within 2 mm. Only gonion showed 2.04 mm in MRE measurements. 

The paired *t*-test showed that there were no significant differences between truth ground and AI prediction on CBCT-LC. No significant differences were observed between the truth ground and AI prediction on MIP-LC. The details are described in Table 3 and Table 4. 

## 4. Discussion 

A fully automated landmark identification system was presented as an alternative option for consistent cephalometric landmark identification in repetitive tasks [30]. Several factors might affect deep learning prediction—the data size, the number of layers in the architecture, computer components, and the image resolution [31,32]. In this study, we intended to train the multi-stage CNNs with two modalities of lateral cephalograms synthesized from CBCT to increase the training samples, to broaden the training ranges, and to enhance the ability of image recognition without additional radiation to the patient.

The purpose of creating and using combination dataset in this study was to perform effective and efficient landmark identification both in manual and AI prediction. Although we used lateral cephalograms synthesized by CBCT to minimize the superimposed and layered bilateral structures, layered images still remained as confounding variables. For instance, the superimposed mandible prominently made two lines, especially in patients who had asymmetry that made it difficult to decipher gonion. Therefore, as a compensation, we tried to use the MIP image to reinforce these defects. However, the paired *t*-test showed that there were no significant differences between truth ground and AI prediction on CBCT-LC, MIP-LC. The AI learned the examiner’s identification pattern from preprocessed image data in deep learning, which explained that there were no significant differences in AI prediction on the CBCT-LC or MIP-LC, in our results. Whether the dataset was combined or not, AI would prediction on the same corresponding position with the same pattern. Details are explained below.

Deep learning is conceptually similar to supervised machine learning, but there are some differences. Supervised machine learning requires that the researcher process the image to extract target features. Deep learning works directly on the data image; and automatically reduces the burden of work. Traditional machine learning should have created a new algorithm for each new datum. On the other hand, retraining in the deep learning model is possible with new data. Substantial number data were used to train the deep learning architecture. Among the deep learning options, CNNs architecture had the outstanding ability to recognize particular appearance patterns that were widely used. This is an essential aspect of cephalometric landmark identification [33,34]. Classic deep CNNs are composed of a convolutional layer, pooling layer, and a fully connected layer. Although CNNs have the outstanding ability to recognize images that are used in medical image science, robustness is limited for geometrical transformations, other than parallel movements, such as scaling and rotation. Therefore, image features with scaling and rotation would present recognition errors in CNNs, but it steadily improved in CNNs architectures.

In this study, we proposed the multi-stage CNNs architecture that was constructed by stacking the convolution layers. The multi-stage architecture consisted of multiple convolutional layers and the number of layers was arranged in increasing parallel order. Since these CNNs structures weights of each convolution layer were connected to share features with corresponding layers of other stages, features of the original input image transferred to each stage. The features of preprocessed image extracted at all stages were concatenated and fulfilled to the integration layer, which showed strengths to improve the accuracy [35,36,37]. 

Most recent systems were developed using a conventional lateral cephalogram. However, the lateral cephalogram synthesized by CBCT had advantages—customization of resolution, ability to re-orientate the image to enhance the image quality, and use of an orthogonal projection to reducing the interference caused by superimposition of bilateral structures. Due to difficulties in the segmentation of internal organs, the MIP algorithm was invented in 1988 by the nuclear medicine doctor Jerold Wallis [38,39]. The MIP image helped to find the relative 3D position of anatomic structures. Emergence of a three-dimensional image and this display technique offered the information on depth and volume. However, since the three-dimensional image required a rendering process, inevitably noise and blurring occurred, inhibiting accurate detection of the anatomic structure. The MIP algorithm provided better detection of the relationship between objects and the surface contours. Structures of interest could be highlighted by selecting the objects, and the surface contour could be indirectly inferred by the depth information. One of the matters with the lateral cephalogram was superimposed bilateral structures, which could be eliminated by the enhanced image contrast in MIP. While MIP was visualized in planar space, it still included a part of the three-dimensional structural information, without using the full three-dimension rendering [39].

**Figure 5:**
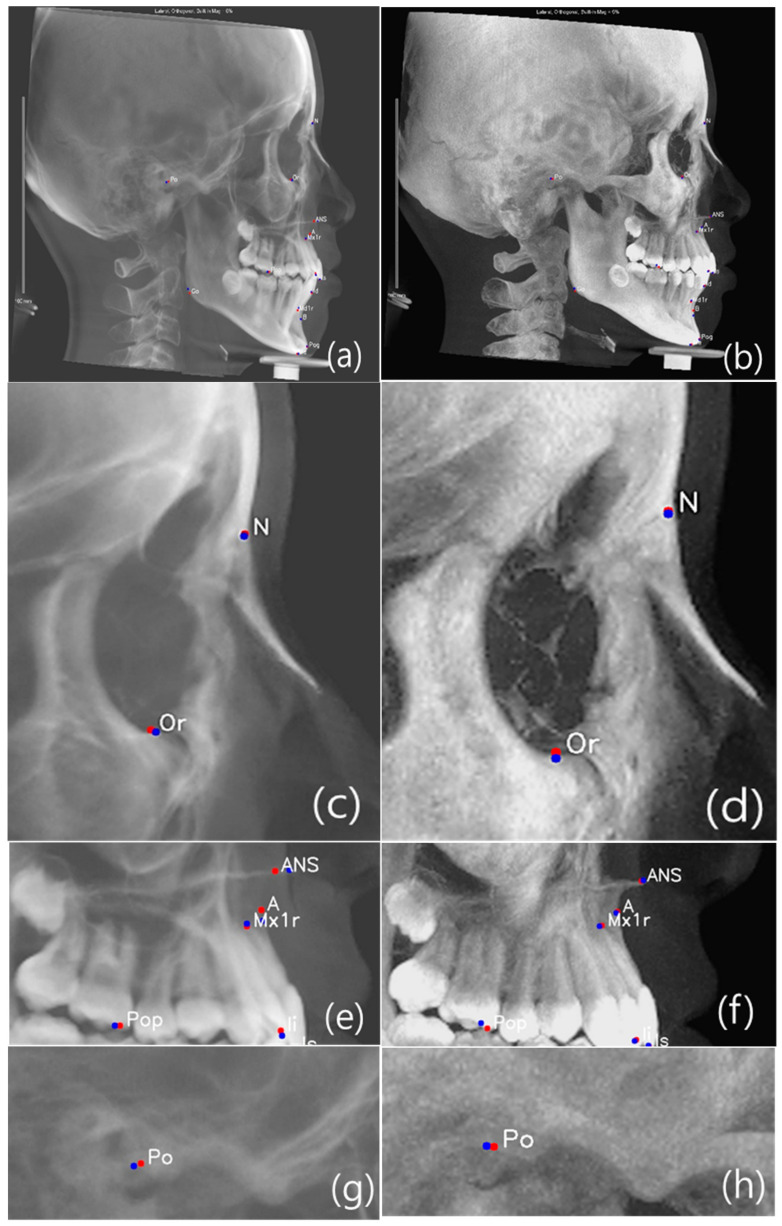

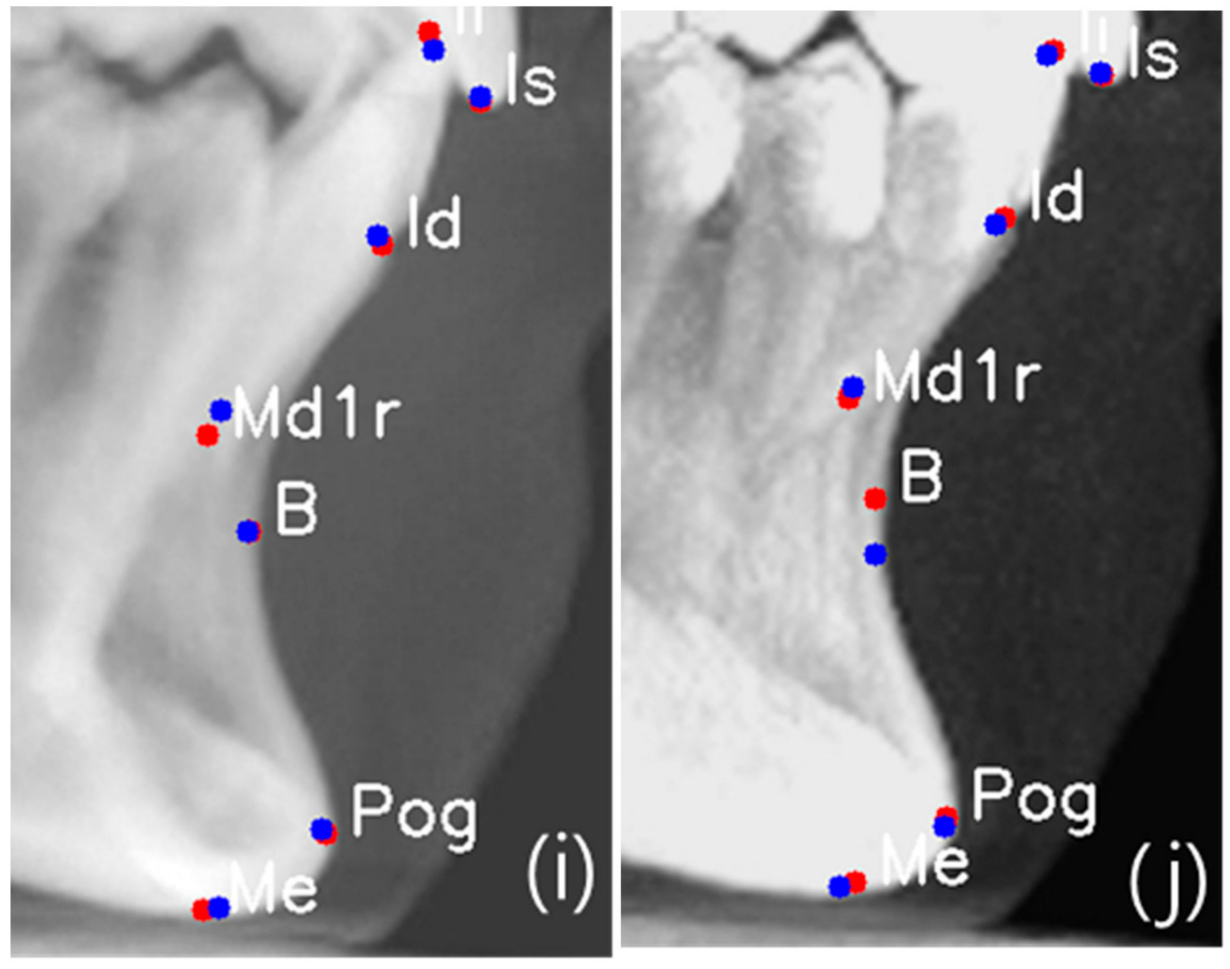
Comparison of AI prediction between CBCT-LC (**a**,**c**,**e**,**g**,**i**) and MIP-LC (**b**,**d**,**f**,**h**,**j**); Orbitale **c** and **d**; Maxilla **e** and **f**; Porion **g** and **h**; and Mandible **i** and **j**. The red dot is manual identification (truth ground), and the blue dot is AI prediction.

Previous studies compared reproducibility between conventional lateral cephalogram and different types of CBCT synthesized 2D lateral cephalograms (MIP, Ray-sum or usual). Conclusion of these studies was that linear and angular measurement derived from CBCT synthesized cephalogram resulted in higher consistency than conventional cephalogram [1,2,6,40,41]. We used CBCT synthesized combination data to perform efficient landmark identification for training. We used 15 landmarks positioned on the anatomic structure contours that were displayed more clearly in the MIP image. Comparison of AI prediction results between CBCT-LC and MIP-LC are shown in Figure 5. 

Although the CBCT was currently used to provide 3D information, further studies would lead to better standardization in cephalometry. The ability of direct landmark identification and measurement was lacking. Even though we obtained the 3D images from patients to use current research-based analyses, it had to be transformed to a 2D aspect [42,43]. 

**Figure 6:**
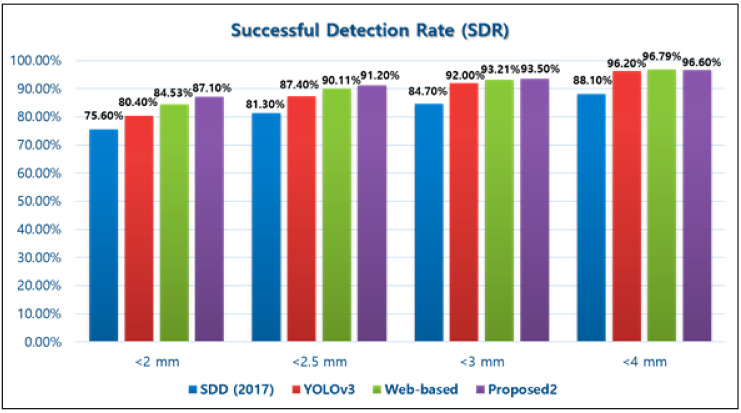
Comparison of successful detection rate of different CNNs architecture based on fully automatic landmark identification system.

In 2017, the first fully-automated cephalometric landmark identification system was applied to a deep CNNs learning model [22]. They trained 400 conventional lateral cephalogram datasets that included 19 landmarks. The result showed a 75.58% SDR range of 0 to 2 mm. In 2019, 1311 trained conventional lateral cephalograms using the You-Only-Look-Once version 3 (YOLOv3) with 80 landmarks achieved an 80.4% SDR range of 0 to 2 mm. The prediction result achieved approximately 5% higher in all ranges of SDR, than a previous study [24]. Another study used a personal computer to develop the CNNs architecture training with 153 lateral cephalograms and tested 66 images. Ten landmarks frequently used in cephalometry were included. There were no significant differences between the manual and automatic prediction in the cephalometric analysis, but the average prediction errors recorded 17.02 in pixel (approximately 4.50 mm) [23]. In 2020, many studies introduced new CNNs algorithms or methods [44,45,46]. Kunz et al. [44] used customized Keras and Tensorflow, similar to us. In their study, MRE was not described but AI prediction showed similar results with examiners. Kim et al. [45] Reported a web-based deep learning method, which had advantage in accessibilities. They evaluated four different dataset groups. The highest SDR range of 0 to 2 mm achieved 84.53 % with an acceptable MRE, 1.37 ± 1.79 mm. Comparison of SDR in different CNNs architecture, based on fully automatic landmark identification systems, is described in Table 5 and Figure 6. However, the deep learning model also applied automatic landmark identification of the CBCT image. Some important factors that affected accuracy for the CNNs-based fully automatic landmark identification system were—CNNs architecture’s structure, sufficient learning data, and the supervising method. Comparison to the above studies, we employed improvements—(i) to achieve better accuracy we increased the number of layers and only extracted the necessary features for learning; (ii) we used a sufficient dataset; and (iii) used combination data to perform efficient supervising (setting the truth ground). The number of landmarks did not significantly affect the system accuracy. However, an increased number of landmarks might enhance clinical procedures. 

The highlights of this study were proposal of customized CNNs architecture and we reported the AI prediction results of CBCT-LC and MIP-LC as a transitional study of 3D CBCT data. However, a limitation of this study, first of all, was that the amount of data required to achieve the expected accuracy could not be explained. A previous study reported that greater the number of data, higher the accuracy [47]. They used 2200 training data as the maximum number to prove it and results of MRE showed more than 1.5 mm. Although we did not try to discover the suitable number of data with a gradual dataset, this was possible to presume through the results, which achieved our expectations, that the means and the amount of data, was used properly in this study. Second, we could not compare if our CNNs architecture was better than that of similar studies as different computer components and dataset were used in other studies. In order to identify whether the combination dataset had any benefit, we needed to compare with a unified dataset in the further study.

## 5. Conclusions

Recently, artificial intelligence technology is rapidly being commercialized. In this study, we introduced a method to customize the CNNs architecture using open-source. A new learning concept in the aspect of the CNNs architecture and dataset achieved superior results compared to previous studies that obtained 87.10% SDR range of 0 to 2 mm with 1.03 mm average MRE. However, no control group was established in this study. Hence, a comparison of CBCT-LC combined with MIP-LC training and CBCT-LC training is needed in future studies, to verify if a combination dataset has any benefit or not.

## Figures and Tables

**Table 1:** Landmark definitions.

Landmarks	Definition
Nasion (N)	The most anterior point of the frontonasal suture
Orbitale (Or)	Inferior margin of the orbit
Porion (Po)	Superior margin of the external auditory meatus
Anterior nasal spine (ANS)	Tip of anterior nasal spine
A point	Point at the deepest concavity on the maxilla between the anterior nasal spine and prosthion
Maxillary central incisor root (Mx1r)	Maxillary central incisor root
Maxillary incisal edge (Is)	Maxillary incisal edge
Mandibular incisal edge (Ii)	Mandibular incisal edge
Infradentale (Id)	The highest and most anterior point on the alveolar process in the median plane between the mandibular central incisors
Mandibular central incisor root (Md1r)	Mandibular central incisor root
B point	Point at the deepest concavity on the mandibular symphysis between infradentale and pogonion
Pogonion (Pog)	The most anterior midpoint of the chin of the mandibular symphysis
Menton (Me)	The most inferior point of the mandibular symphysis
Gonion (Go)	Most posterior inferior point on angle of mandible
Posterior occlusal plane point (Pop)	Posterior occlusal plane point: mesio-buccal cusp of 1st molar

**Table 2:** System prediction results of 15 landmark identification in combination data.

Landmark.	MRE (mm)	SD (mm)	SDR (%)			
			2.0 mm	2.5 mm	3.0 mm	4.0 mm
N	0.56	1.497	95.97	96.55	97.12	98.27
Or	0.69	1.397	97.12	98.27	98.27	98.85
Po	1.43	1.689	78.73	84.48	89.65	94.82
ANS	0.76	0.726	93.67	97.12	98.27	99.42
A	1.22	2.697	89.08	91.95	96.55	98.27
Mx1r	1.59	1.308	69.54	78.73	86.2	95.4
Is	0.61	1.081	93.1	94.25	94.82	98.27
Id	0.79	1.080	90.8	94.25	95.4	96.55
Ii	0.65	0.927	93.67	94.25	95.97	97.7
Md1r	1.39	1.266	81.03	88.5	89.65	94.25
B	1.09	0.891	83.33	92.52	95.97	98.85
Pog	0.58	0.580	95.97	97.7	98.85	100
Me	0.59	0.547	97.12	98.27	98.85	100
Go	2.04	1.727	62.64	72.98	77.58	85.63
Pop	1.31	1.897	85.05	87.93	89.65	92.52
Average	1.03	1.288	87.13	91.19	93.52	96.59

**Table 3:** Comparison of landmark identification between truth ground and AI prediction on the CBCT synthesized lateral cephalograms (CBCT-LC).

	Truth Ground				AI Prediction CBCT-LC				*p*-Value *
Landmark	Mean	SD	Min	Max	Mean	SD	Min	Max	
N_x	1642.71	58.46	1479.00	1866.00	1641.33	55.81	1487.66	1849.16	0.868
N_y	564.09	124.36	285.00	865.00	551.58	122.73	259.93	828.51	0.488
Or_x	1561.92	55.80	1363.00	1755.00	1558.83	54.22	1378.06	1732.80	0.703
Or_y	819.48	105.41	628.00	1079.00	805.06	105.67	604.01	1047.61	0.350
Po_x	851.02	81.24	526.00	991.00	850.27	78.83	530.84	989.41	0.950
Po_y	827.38	107.98	633.00	1085.00	819.01	107.73	612.38	1088.11	0.593
ANS_x	1665.24	55.63	1505.00	1875.00	1663.51	52.62	1497.87	1838.49	0.829
ANS_y	1052.13	86.66	883.00	1280.00	1042.43	90.04	834.11	1275.94	0.453
A_x	1642.36	54.26	1496.00	1851.00	1640.73	52.12	1497.96	1831.31	0.833
A_y	1105.75	84.83	946.00	1327.00	1095.83	88.24	914.74	1325.59	0.423
Mx1r_x	1618.64	53.64	1468.00	1813.00	1612.80	50.99	1479.12	1779.62	0.457
Mx1r_y	1134.80	79.14	969.00	1349.00	1128.02	79.84	951.91	1323.55	0.545
Is_x	1697.26	68.22	1534.00	1926.00	1697.33	65.10	1543.72	1899.46	0.994
Is_y	1314.39	75.45	1150.00	1499.00	1306.30	78.15	1123.21	1506.45	0.455
Id_x	1655.10	79.85	1475.00	1873.00	1650.89	75.27	1482.44	1848.20	0.716
Id_y	1392.60	64.48	1262.00	1546.00	1384.93	70.47	1224.56	1557.32	0.401
Ii_x	1677.85	75.42	1515.00	1902.00	1677.02	70.94	1514.51	1875.32	0.939
Ii_y	1305.05	69.12	1164.00	1475.00	1294.47	72.27	1129.55	1466.30	0.282
Md1r_x	1596.45	82.52	1414.00	1788.00	1590.17	78.16	1415.73	1778.64	0.605
Md1r_y	1453.87	58.29	1336.00	1575.00	1445.50	64.19	1283.63	1621.69	0.326
B_x	1607.09	86.88	1424.00	1813.00	1602.59	82.05	1424.10	1785.40	0.723
B_y	1506.37	64.14	1337.00	1628.00	1497.51	71.16	1294.64	1650.67	0.358
Pog_x	1613.85	93.19	1419.00	1837.00	1610.07	88.90	1419.37	1809.18	0.785
Pog_y	1601.34	58.20	1471.00	1704.00	1600.11	58.44	1442.15	1712.63	0.880
Me_x	1555.66	96.74	1352.00	1783.00	1553.77	91.11	1355.66	1748.78	0.894
Me_y	1663.93	55.23	1526.00	1770.00	1658.06	55.99	1510.33	1773.85	0.445
Go_x	992.75	87.06	725.00	1184.00	988.62	79.60	719.04	1152.65	0.748
Go_y	1373.97	65.90	1202.00	1537.00	1355.27	64.39	1213.18	1528.06	0.053
Pop_x	1434.15	58.52	1264.00	1616.00	1425.49	56.55	1261.20	1588.02	0.314
Pop_y	1263.29	65.40	1130.00	1426.00	1253.58	68.57	1104.16	1427.76	0.292

* Paired *t*-test was done for comparison of manual identification and AI prediction on CBCT-LC. Unit of measurement: Pixel.

**Table 4:** Comparison of landmark identification between truth ground and AI prediction on CBCT synthesized MIP lateral cephalograms (MIP-LC).

	Truth Ground				AI prediction MIP-LC				*p*-Value *
Landmark	Mean	SD	Min	Max	Mean	SD	Min	Max	
N_x	1642.71	58.46	1479.00	1866.00	1646.17	59.46	1479.27	1862.75	0.754
N_y	564.09	124.36	285.00	865.00	562.05	118.36	288.38	830.26	0.793
Or_x	1561.92	55.80	1363.00	1755.00	1565.48	57.18	1369.46	1756.59	0.649
Or_y	819.48	105.41	628.00	1079.00	819.07	100.80	630.06	1048.80	0.959
Po_x	851.02	81.24	526.00	991.00	850.48	81.05	517.92	986.90	0.920
Po_y	827.38	107.98	633.00	1085.00	822.26	101.70	635.18	1053.43	0.156
ANS_x	1665.24	55.63	1505.00	1875.00	1670.20	56.72	1505.48	1875.80	0.406
ANS_y	1052.13	86.66	883.00	1280.00	1052.22	86.83	875.05	1278.58	0.752
A_x	1642.36	54.26	1496.00	1851.00	1645.53	55.47	1496.60	1849.64	0.778
A_y	1105.75	84.83	946.00	1327.00	1107.52	83.06	950.80	1328.57	0.752
Mx1r_x	1618.64	53.64	1468.00	1813.00	1621.62	54.20	1479.40	1825.12	0.771
Mx1r_y	1134.80	79.14	969.00	1349.00	1138.54	77.21	980.84	1351.76	0.333
Is_x	1697.26	68.22	1534.00	1926.00	1701.67	68.60	1542.86	1927.34	0.601
Is_y	1314.39	75.45	1150.00	1499.00	1314.79	74.57	1149.57	1501.82	0.599
Id_x	1655.10	79.85	1475.00	1873.00	1655.84	78.72	1482.70	1873.20	0.799
Id_y	1392.60	64.48	1262.00	1546.00	1397.29	65.69	1262.71	1561.40	0.381
Ii_x	1677.85	75.42	1515.00	1902.00	1679.90	74.65	1512.09	1899.68	0.986
Ii_y	1305.05	69.12	1164.00	1475.00	1307.15	69.11	1164.50	1474.31	0.770
Md1r_x	1596.45	82.52	1414.00	1788.00	1596.19	82.44	1408.86	1815.76	0.684
Md1r_y	1453.87	58.29	1336.00	1575.00	1456.71	62.78	1310.59	1610.44	0.997
B_x	1607.09	86.88	1424.00	1813.00	1608.83	85.34	1422.78	1812.24	0.979
B_y	1506.37	64.14	1337.00	1628.00	1507.28	67.61	1326.61	1642.09	0.435
Pog_x	1613.85	93.19	1419.00	1837.00	1615.02	91.87	1415.98	1837.82	0.915
Pog_y	1601.34	58.20	1471.00	1704.00	1607.11	57.09	1467.66	1703.10	0.294
Me_x	1555.66	96.74	1352.00	1783.00	1556.17	94.12	1353.87	1767.15	0.862
Me_y	1663.93	55.23	1526.00	1770.00	1666.42	55.77	1522.65	1768.89	0.657
Go_x	992.75	87.06	725.00	1184.00	992.71	81.71	719.39	1169.11	0.941
Go_y	1373.97	65.90	1202.00	1537.00	1374.04	63.35	1186.13	1531.91	0.493
Pop_x	1434.15	58.52	1264.00	1616.00	1429.53	60.57	1253.62	1616.57	0.064
Pop_y	1263.29	65.40	1130.00	1426.00	1261.56	65.56	1131.64	1427.02	0.182

* Paired *t*-test was done for comparison of manual identification and AI prediction on CBCT-MIP. Unit of measurement: Pixel.

**Table 5:** Comparison of automatic landmark identification system prediction results.

Method.	Landmark	Total Data	SDR (%)			
			2.0 mm	2.5 mm	3.0 mm	4.0 mm
SDD^22^	19	400	75.6	81.3	84.7	88.1
YOLOv3^24^	80	1311	80.4	87.4	92.0	96.2
Web-based^45^	23	2075	84.5	90.1	93.2	96.8
Proposed	15	860	87.1	91.2	93.5	96.6

## Data Availability

Data sharing not applicable.
